# The Impact of Viral Concentration Method on Quantification and Long Amplicon Nanopore Sequencing of SARS-CoV-2 and Noroviruses in Wastewater

**DOI:** 10.3390/microorganisms13020229

**Published:** 2025-01-22

**Authors:** George Scott, Nicholas P. Evens, Jonathan Porter, David I. Walker

**Affiliations:** 1 Centre for Environment, Fisheries and Aquaculture Science, The Nothe, Barrack Road, Weymouth DT4 8UB, UK; 2 Environment Agency, National Monitoring, Starcross, Exeter EX6 8FD, UK

**Keywords:** wastewater, SARS-CoV-2, norovirus, viral concentration, wastewater surveillance, crAssphage, qPCR, nanopore sequencing, long amplicon, whole-genome sequencing

## Abstract

Wastewater-based surveillance has gained attention in the four years following the start of the COVID-19 pandemic. Accurate pathogen detection, quantification and characterisation rely on the selection of appropriate methodologies. Here, we explore the impact of viral concentration method on RT-qPCR inhibition and quantification of norovirus genogroups I and II (GI and GII), crAssphage, phi6 and SARS-CoV-2. Additionally, their impact on long amplicon sequencing for typing noroviruses and whole-genome sequencing (WGS) SARS-CoV-2 was explored. RT-qPCR inhibition for each viral concentration method was significantly different apart from the two ultrafiltration methods, InnovaPrep^®^ concentrating pipette (IP) and Vivaspin^®^ (VS) centrifugal concentrators. Using an ultrafiltration method reduced inhibition by 62.0% to 96.0% compared to the ammonium sulphate (AS) and polyethylene glycol (PEG) precipitation-based methods. Viral quantification was significantly impacted by concentration method with the highest concentrations (copies/L) observed for VS with 7.2- to 83.2-fold differences from AS depending on the target. Norovirus long amplicon sequencing showed genotype-dependent differences with IP performing best for GI and VS for GII although IP performance gains for GI were relatively small. VS outperformed AS and IP across all metrics during SARS-CoV-2 WGS. Overall, VS performed the best when considering all the areas of investigation.

## 1. Introduction

Wastewater-based surveillance (WBS) became an invaluable tool during the COVID-19 pandemic following the detection of SARS-CoV-2 in faeces, with 72 countries adopting WBS to monitor the pandemic [1,2]. The success of WBS, however, is dependent on employing the correct methods throughout the workflow from sample collection onwards. Method development and optimisation has stemmed through all areas and has included, but not been limited to, studies investigating the location and timing of sampling to create representative data, sample collection techniques, viral particle and nucleic acid concentration, nucleic acid extraction, viral quantification, viral genome sequencing, variant identification and data normalisation and analysis [3,4,5,6,7,8,9,10,11]. In this study, we focused on viral concentration methods.

Due to low titres of viral nucleic acids in wastewater, concentration of viral particles or nucleic acids is usually required for WBS. Successful quantification of SARS-CoV-2 RNA from wastewater has been used to predict the prevalence of SARS-CoV-2 in the community and to forecast disease [12,13,14]. Many studies on viral particle concentration methods have been undertaken [15,16,17,18,19,20,21,22,23,24,25,26,27,28,29]. Common viral particle concentration methods include adsorption–elution with charged membranes; bag-mediated filtration systems (BMFSs); capture using porous nanoparticles; skimmed milk flocculation (SMF); precipitation with polyethylene glycol (PEG), aluminium chloride or ammonium sulphate (AS); ultrafiltration (using centrifugation or pressure) and ultracentrifugation. The co-concentration of free viral nucleic acids during these procedures should not be disregarded. Direct concentration of nucleic acids from small (≈1 mL) and large (≈40 mL) wastewater samples has also been investigated but to a lesser extent [30,31,32,33,34].

The previous studies have focused on viral quantification using RT-qPCR, -dPCR or -ddPCR using assays targeting SARS-CoV-2, SARS-CoV-2 surrogates, process controls and biomarkers for population normalisation. Recently, the impact of viral concentration method on the quantification of adenovirus, astrovirus, influenza A and B, measles virus, norovirus genogroups I (GI) and II (GII) and rotavirus has been explored [23,33]. Sequencing has also been adopted in WBS to identify and track variants of concern and emerging variants [35,36]. Five studies have explored the impact of viral concentration method using Illumina-based sequencing, three of which used metagenomic approaches [16,29,34] and two used short amplicon PCR tiling-based approaches [28,33].

In recent years, the interest in long-read sequencing methods has grown, partly due to its ability to resolve repeated genomic regions, identify post-transcriptional modifications and provide improved phylogenetic resolution through metabarcoding [37,38,39,40]. Few WBS methods, however, have been developed that fully utilise long-read sequencing. The 400 bp ARTIC panel for SARS-CoV-2 was originally developed for use with Oxford Nanopore sequencing and has been applied on several occasions [36,41,42]. As the ARTIC amplicons are a suitable length for shorter-read sequencing, Illumina sequencing was often employed. A ≈1200 bp PCR-tiling SARS-CoV-2 whole-genome sequencing (WGS) assay was developed [43] and later applied to wastewater with Oxford Nanopore sequencing [44]. This reduced the number of amplicons from 99 to 29 while still achieving 97% genome coverage. Recently, a long amplicon method for sequencing norovirus GI and GII in wastewater was developed [45]. A ≈1000 bp region containing partial *VP1* and *RdRp* regions was amplified, sequenced using Oxford Nanopore and then typed using the norovirus dual-typing system [46].

The aim of this study was to perform comparative analysis of several viral concentration methods with a focus on SARS-CoV-2 and norovirus. The impact of concentration method on RT-qPCR inhibition and viral quantification was assessed along with long amplicon sequencing for typing noroviruses and WGS of SARS-CoV-2.

## 2. Materials and Methods

### 2.1. Sampling, Viral Concentration and Nucleic Acid Extraction

A total of 240 untreated wastewater samples were collected by the Environment Agency (Exeter, UK) between 28 October and 20 November 2021 as part of the Environmental Monitoring for Health Protection programme in England. Briefly, 1 L samples were collected and concentrated using ammonium sulphate (AS) and polyethylene glycol (PEG) precipitation and two ultrafiltration-based methods using 10 kDa Vivaspin^®^ (VS) centrifugal concentrators (Sartorius, Göttingen, Germany) and an InnovaPrep^®^ (Drexel, MO, USA) concentrating pipette with 0.05 µm filter tips (IP). For all methods, initial sample clarification was performed by centrifugation at 10,000× *g* for 30 min at 4 °C using 200 mL for AS and PEG and 50 mL for VS and IP. Clarified wastewater was spiked with 1 mL phi6 process control per 150 mL [47].

For AS, 150 mL of clarified wastewater was mixed with 60 g of ammonium sulphate and incubated for ≥1 h at 4 °C. For PEG, 150 mL of clarified wastewater was adjusted to pH 7.0 to 7.6 with NaOH and added to 50 mL of 40% (*w*/*v*) PEG 8000, 8% (*w*/*v*) NaCl solution, inverted several times to mix and incubated at 4 °C overnight. PEG and AS were then centrifuged at 10,000× *g* for 30 min at 4 °C and the supernatant discarded. For IP, 25 mL of clarified wastewater was spiked with 250 µL of 5% (*v*/*v*) Tween 20, processed using a 0.05 µm tip and then eluted. For VS, 15 mL of clarified wastewater was added to the spin column and centrifuged for 40 min at 4500× *g* at 4 °C or until the sample volume was ≤0.5 mL. Two millilitres of NucliSENS^®^ lysis buffer (BioMérieux, Lyon, France) was added to the PEG and AS pellets, into the IP eluate and onto the VS membrane and incubated for 10 min. Nucleic acid extraction was performed on a KingFisher™ Flex (Thermo Fisher Scientific, Waltham, MA, USA) using NucliSENS^®^ reagents (BioMérieux) [47].

### 2.2. Viral Quantification and RT-qPCR Inhibition

Nine-hundred and sixty nucleic acid extracts from the 240 wastewater samples processed using the four different viral concentration methods were used for the quantification of SARS-CoV-2, crAssphage and phi6. SARS-CoV-2 and phi6 were quantified using RT-qPCR and run alongside an ssRNA standard curve using RNA Ultramer™ oligonucleotides (Integrated DNA Technologies, Coralville, IA, USA) [47]. crAssphage qPCRs were run with a dsDNA standard curve using Ultramer™ DNA oligonucleotides (Integrated DNA Technologies) (Supplementary Methods S1). SARS-CoV-2 quantities were not normalized based on phi6 recoveries.

Eighty nucleic acid extracts representing 20 wastewater samples processed using the four viral concentration methods were sent on dry ice to the Centre for Environment, Fisheries and Aquaculture Science (Weymouth, UK) and stored at −80 °C upon arrival. These samples were used for determining RT-qPCR inhibition and quantification of noroviruses GI and GII. Due to the lack of nucleic acid volume, PEG processed samples were not used for norovirus quantification or sequencing. Only ten samples had sufficient nucleic acid volume across each of the viral concentration methods for norovirus and SARS-CoV-2 long amplicon sequencing.

To measure the influence of viral concentration method on the carryover of wastewater constituents that impact polymerase and reverse transcriptase efficiency, an RT-qPCR inhibition assay was employed using an external control RNA method [48]. Samples were spiked with 1 µL of external control RNA to a concentration of ≈200,000 copies/µL and run alongside an external control RNA + water control reaction to allow calculation of inhibition [48]. Samples with inhibition levels <0% were assigned as 0%. For quantification of GI and GII, a duplex one-step RT-qPCR was used and run alongside a ssRNA standard curve using RNA Ultramer™ oligonucleotides (Integrated DNA Technologies) [49]. For all RT-qPCR and qPCRs, samples were run in duplex and a 5-point, 10-fold dilution standard curve was used starting at 100,000 copies/µL. Quality control thresholds of the standard curve slopes were between −3.6 and −3.1 with an R^2^ of >0.98.

### 2.3. Norovirus Long Amplicon Sequencing

Long amplicon sequencing was used to type norovirus GI and GII [45]. Briefly, nucleic acid extracts were cleaned with Mag-Bind^®^ TotalPure NGS beads (Omega Bio-Tek, Norcross, GA, USA) and cDNA was synthesised using LunaScript^®^ RT SuperMix (New England Biolabs, Ipswich, MA, USA). Semi-nested PCR using Platinum™ Taq polymerase (Invitrogen™, Waltham, MA, USA) was used to amplify GI or GII in isolation. PCR controls were checked using a TapeStation 4150 using D5000 screen tape (Agilent, Santa Clara, CA, USA). PCR products were cleaned using ExoSAP-IT™ (Applied Biosystems, Waltham, MA, USA), quantified using the Qubit HS dsDNA kit (Invitrogen™) and pooled (85.3 and 114.7 fmol for GI and GII). Barcoding was performed using the Native Barcoding Kit 96 V14 (Oxford Nanopore Technologies, Oxford, UK). Libraries were sequenced on R10.4.1 flow cells using a Mk1C with MinKNOW version 23.07.12 until ≈60,000 reads were collected per barcode. Data were basecalled using super accuracy basecalling on MinKNOW version 24.02.10.

A consensus-based bioinformatics approach was used. Reads were split, trimmed and size-filtered and then randomly sampled to screen for chimeras and generate consensus sequences. Consensus sequences were polished, screened for regions with poor support, clustered at 95% and typed. Reads were then aligned against consensus sequences. Full sequencing and bioinformatic procedures can be found on protocols.io dx.doi.org/10.17504/protocols.io.8epv5xpmjg1b/v2.

### 2.4. SARS-CoV-2 Long Amplicon Whole-Genome Sequencing

Nucleic acids were cleaned and cDNA was synthesised as above. A PCR tiling approach using the V2 primers was used for WGS SARS-CoV-2 [43]. Briefly, two PCR reactions were run per sample, each containing primers from alternating amplicons. Q5^®^ Hot Start HF 2x Master Mix (New England Biolabs) was used with a 25 µL total volume and 2.5 µL of template. PCR control analysis, product cleanup and quantification, barcoding, sequencing and basecalling were performed as above with the following changes. Equal volumes of PCR products from both tiling reactions were pooled, the volume of end-prepped DNA input into barcoding was increased to 3 µL and sequencing was performed for ≈36 h with a flow cell wash and additional library aliquot loaded after 18 h. Bioinformatics are outlined in Table 1.

Briefly, reads were split, primers were trimmed and trimmed reads were then filtered for quality (>Q20) and length (900 to 1500 bp). Trimmed and filtered reads were aligned to the SARS-CoV-2 Wuhan-Hu-1 reference genome (Accession NC_045512.2). Sequencing depth was calculated using IGV with measurements taken at the mid-point from each of the 29 amplicons. Read depth thresholds were adjusted for each amplicon individually to account for barcode bleeding identified in the negative control. A read depth threshold of 10 was used to determine coverage.

### 2.5. Data Analysis

Comparisons of RT-qPCR detection rates were only performed on samples where data were available for each of the different viral concentration methods. For comparison of viral quantification, only samples where a quantitative measurement was available for each of the concentration methods were analysed. To compare the overall performance of the different concentration methods, scores of 3, 2 or 1 were assigned based on the performance of each of the areas under assessment. Statistical analyses were performed in R using R Studio build 353 (version 2022.12.0) using the rstatix package (version 0.7.2) [56]. For inferential statistics, a repeated measure ANOVA was performed unless the assumption of normality was not met, where a Friedman test was performed. Post hoc testing was performed using paired *t*-test or Wilcoxon signed-rank test both with Bonferroni correction. Statistical significance was defined as *p* < 0.05.

## 3. Results

### 3.1. RT-qPCR Inhibition and Viral Quantification

**Figure 1:**
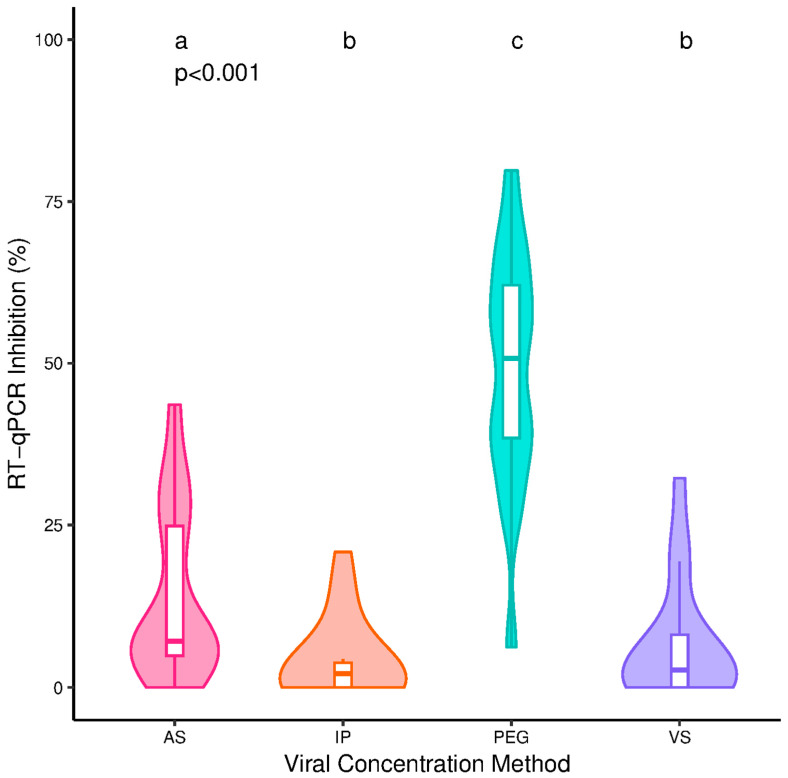
The RT-qPCR inhibition from the SARS-CoV-2 N1 assay from samples processed using different viral concentration techniques: ammonium sulphate (AS), InnovaPrep (IP), (VS) Vivaspin and polyethylene glycol (PEG). *p*-values reported are from the initial hypothesis testing. Groups within a subfigure sharing the same lettering are not significantly different from each other following pairwise comparison (*p* > 0.05). N = 17.

**Figure 2:**
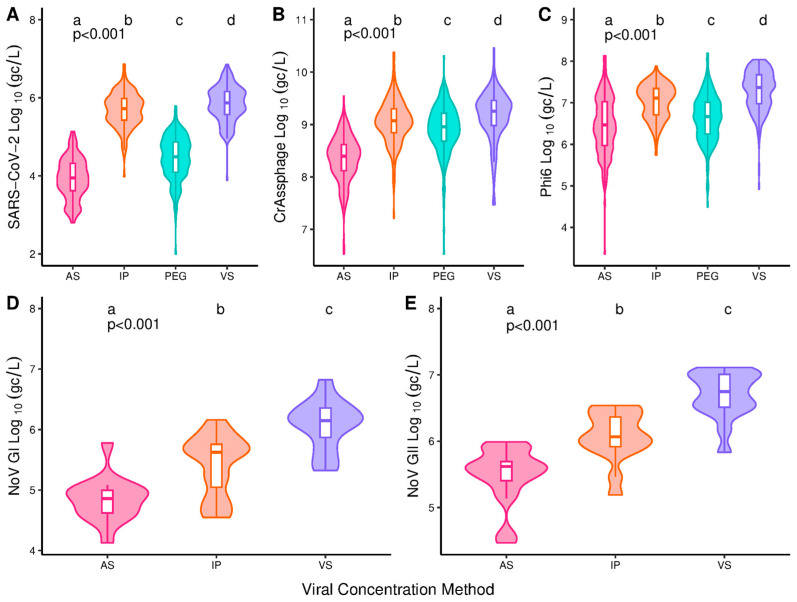
SARS-CoV-2 (**A**), crAssphage (**B**), phi6 (**C**), norovirus genogroup I (**D**) and norovirus genogroup II (**E**) concentrations from wastewater samples processed using different viral concentration techniques: ammonium sulphate (AS), InnovaPrep (IP), polyethylene glycol (PEG) and (VS) Vivaspin. *p*-values reported are from the initial hypothesis testing. Groups within a subfigure sharing the same lettering are not significantly different from each other following pairwise comparison (*p* > 0.05). N = 189 (**A**), 226 (**B**), 236 (**C**), 15 (**D**) and 17 (**E**).

Concentration method had a significant impact (*p* < 0.001) on RT-qPCR inhibition with significant pairwise differences between each of the methods apart from VS and IP (Figure 1). Median inhibition was highest for PEG (50.7%), followed by AS (7.1%), VS (2.7%) and IP (2.1%). Quantification of SARS-CoV-2, crAssphage, phi6, and norovirus GI and GII were all significantly impacted by concentration method (*p* < 0.001), with all methods significantly different from each other (Figure 2). AS produced the lowest viral concentrations for all targets with medians of 3.95, 8.40, 6.46, 4.86 and 5.62 log10 (copies/L) for SARS-CoV-2, crAssphage, phi6 and noroviruses GI and GII, respectively. VS produced the highest medians of 5.87, 9.26, 7.36, 6.15 and 6.75 log10 (copies/L) for SARS-CoV-2, crAssphage, phi6 and noroviruses GI and GII, respectively (Figure 2). Correcting SARS-CoV-2 concentrations using RT-qPCR inhibition did not influence the methods’ relative performance (Supplementary Figure S1).

**Figure 3:**
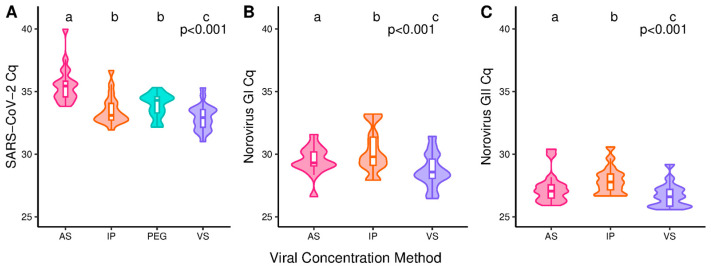
The cycle of quantification (Cq) values for SARS-CoV-2 (**A**), norovirus GI (**B**) and norovirus GII (**C**). Samples were processed using different viral concentration techniques: ammonium sulphate (AS), polyethylene glycol (PEG), InnovaPrep (IP) and (VS) Vivaspin. *p*-values reported are from the initial hypothesis testing. Groups within a subfigure sharing the same lettering are not significantly different from each other following pairwise comparison (*p* > 0.05). N = 19 (**A**), 15 (**B**) and 17 (**C**).

Viral concentration method had a significant impact on RT-qPCR Cq (*p* < 0.001) for all the target pathogens. VS has significantly lower Cqs when compared to each of the other methods under investigation (Figure 3A–C). For SARS-CoV-2, median Cqs were 32.9, 33.1, 34.3 and 35.4 for VS, IP, PEG and AS (Figure 3A). For GI, the median Cqs were 28.6, 29.3 and 29.8 for VS, AS and IP while for GII they were 26.5, 27.1 and 27.8 (Figure 3B,C). For SARS-CoV-2, the detection rates were lowest for the AS method (84.75%) and PEG (90.68%) and highest for IP (99.58%) and VS (97.03%) (Supplementary Table S1). For norovirus GI and GII, all samples were positive for all the concentration methods used. For crAssphage and phi6, a single sample was negative for the VS methods.

### 3.2. Norovirus Long Amplicon Sequencing

**Figure 4:**
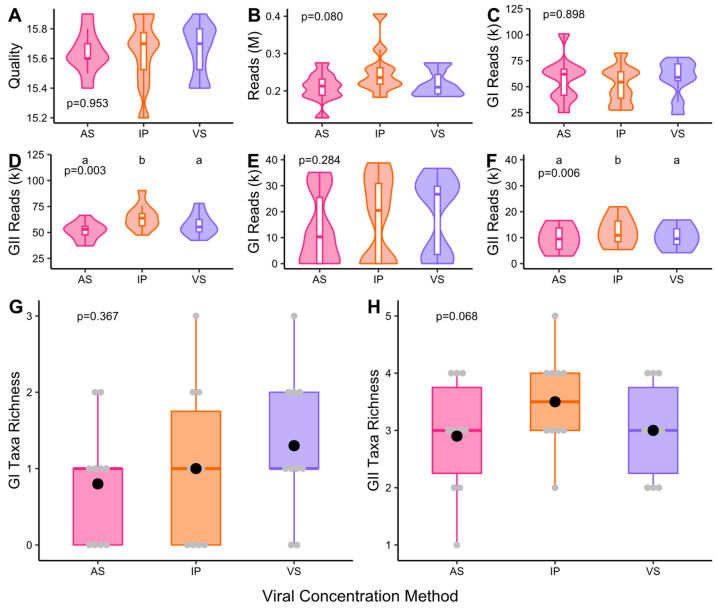
Norovirus long amplicon sequencing data from samples processed using different viral concentration techniques; ammonium sulphate (AS), InnovaPrep (IP) and Vivaspin (VS). Figures show (**A**) median read quality, (**B**) total read count, (**C**) GI trimmed reads, (**D**) GII trimmed reads, (**E**) GI aligned reads, (**F**) GII aligned reads, (**G**) GI taxa richness and (**H**) GII taxa richness. For (**G**,**H**), the grey markers show the raw taxa richness values and the black markers show the group average. *p*-values reported are from the initial hypothesis testing. Groups within a subfigure sharing the same lettering are not significantly different from each other following pairwise comparison (*p* > 0.05). N = 10.

PCR no-template controls were negative, but 985 reads were obtained following sequencing. After primer trimming, 221 and 202 reads remained for GI and GII; none aligned to consensus sequences. Concentration method did not impact read quality (*p* = 0.953), number of raw reads (*p* = 0.80) or GI trimmed reads (*p* = 0.898) (Figure 4A–C). For GII, concentration method significantly impacted trimmed reads (*p* = 0.003) with IP at 63.8 k compared to 53.0 k for AS and 55.3 k for VS. Aligned reads were not significantly impacted for GI, but median aligned read values were not in proximity at 10.3, 20.5 and 26.7k for AS, IP and VS (Figure 4E).

For GII, concentration method significantly impacted aligned reads with IP at 10.9 k compared to 9.4 k for AS and 9.6 k for VS (Figure 4F). Concentration method did not significantly impact taxa richness for GI (*p* = 0.367) or GII (*p* = 0.068). Mean taxa richness was 0.8, 1.0 and 1.3 for GI and 2.9, 3.5 and 3.0 for GII for AS, IP and VS, respectively (Figure 4G,H). A total of seven norovirus GI types were detected; six were detected by VS and five by AS and IP (Supplementary Table S2). The same six GII types were detected by each of the different concentration methods (Supplementary Table S3).

### 3.3. SARS-CoV-2 Long Amplicon Whole-Genome Sequencing

**Figure 5:**
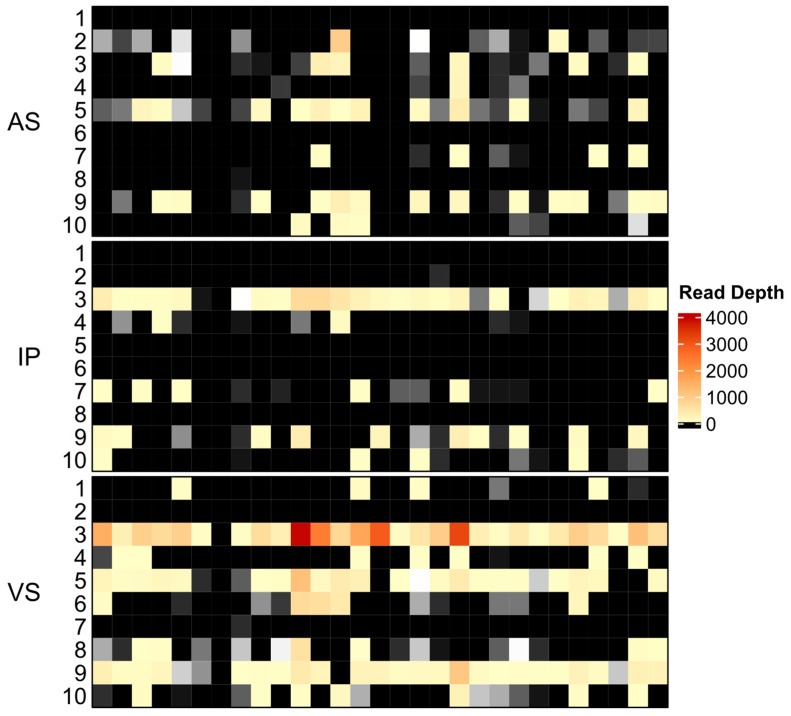
Read depth for each of the 29 SARS-CoV-2 amplicons (columns) that comprise the 1200 bp amplicon panel used for long amplicon sequencing of SARS-CoV-2. Samples were processed using different viral concentration techniques: ammonium sulphate (AS), InnovaPrep (IP) and Vivaspin (VS). Black indicates no reads were aligned to the reference genomes. Grey indicates reads aligned to a given amplicon were <10. Yellow to red indicates that read depth was ≥10 and ≤4000. N = 10.

PCR no-template controls were negative as assessed via TapeStation analysis (Supplementary Figure S2). Sequencing produced 36,225 reads with 753 remaining after quality and length filtering and primer trimming with 713 aligning across the 29 amplicons. This is likely due to barcode bleeding during library prep due to the failure to completely terminate barcode ligation. Read depths were adjusted accordingly (Supplementary Table S4). Overall, coverage and read depth was poor across the viral concentration techniques with five, three and two samples having no regions with >10× read depth for IP, AS and VS (Figure 5). For VS, 80% of samples had >10% coverage compared to 60% and 40% or AS and IP. No AS or IP samples had coverage >80%, whereas 20% of samples had >80% coverage for VS (Figure 5).

**Figure 6:**
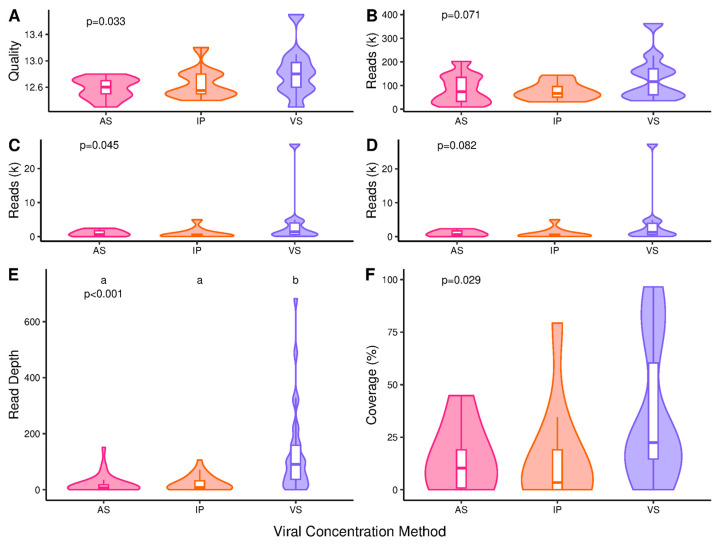
SARS-CoV-2 long amplicon sequencing data from samples processed using different viral concentration techniques; ammonium sulphate (AS), InnovaPrep (IP) and Vivaspin (VS). Figures show (**A**) median read quality, (**B**) the total read count, (**C**) primer trimmed, Q20 and length-filtered reads, (**D**) reference-genome-aligned reads, (**E**) average read depth per amplicon and (**F**) coverage. *p*-values reported are from the initial hypothesis testing. Groups within a subfigure sharing the same lettering are not significantly different from each other following pairwise comparison (*p* > 0.05). N = 10 for (**A**–**D**,**F**) and 29 for (**E**).

Viral concentration significantly impacted median read quality (*p* = 0.033) with median scores of 12.8, 13.2 and 13.7 for AS, IP and VS; no pairwise differences were identified (Figure 6A). The number of raw reads was not significantly impacted (*p* = 0.071) but was highest for VS at 116.3k compared to 74.1 and 67.2k for AS and IP (Figure 6B). A significant impact was identified for the Q20, length and primer trimmed reads (*p* = 0.045), but no pairwise differences were identified; these were 626.0, 356.5 and 1424.0 for AS, IP and VS (Figure 6C). The number of aligned reads was not significantly impacted, but VS method produced the greatest number at 1300.5 compared to 597.5 and 310.0 for AS and IP (Figure 6D). Median read depth per amplicon was significantly impacted (*p* < 0.001) with VS at 91.0 and AS and IP at 6.9 and 7.6 (Figure 6E). Median assay coverage was significantly impacted (*p* = 0.029) at 10.3% 3.5% and 22.4% for AS, IP and VS; although no pairwise differences were identified (Figure 6F).

## 4. Discussion

### 4.1. RT-qPCR Inhibition

Although much work has been performed on the influence of viral concentration method on quantification, little insight into their impact on RT-PCR inhibition has been provided. To date, few studies have performed comparative inhibition testing across different viral concentration methods, fewer have reported the data and none have undertaken quantitative assessment using an assay targeting the WBS pathogen of interest. Of the limited information available, data are variable with reports of no significant inhibition [17,18] to inhibition across all methods [16,19] with studies inferring inhibition from changes in detection rates and Cq following 10-fold dilution of samples.

Understanding the quantitative impact of RT-qPCR inhibition, however, is important, as intra-method inhibition levels were shown to vary from 0 to 98% for AS [48]. Here, we show that ultrafiltration-based methods reduce RT-qPCR inhibition by 62.0% to 96.0% (Figure 1). Some caution, however, should be used when interpreting the data due to the following factors. First, RT-qPCR inhibition is dependent on assay [57] and reagent [48] and only the SARS-CoV-2 N1 assay using qScript XLT 1-Step RT-qPCR ToughMix was investigated here. Second, these data should be used within the context of this study and not for inferring the maximum levels of inhibition to be observed in a WBS programme. Due to the physiochemical heterogeneity of wastewater, it is possible that higher and more variable levels of inhibition would be observed when adopting these methods in a large-scale WBS scheme.

The increased RT-qPCR inhibition observed for the AS and PEG methods could be due to several reasons. First, precipitation–solution components are known to inhibit PCR [58,59,60] and carry-over may further increase inhibition. Second, wastewater and precipitation solution component volumes carried over into lysis may increase or be more variable for the precipitation-based methods. Third, a greater total mass of inhibitors may be present in the sample for precipitation methods (150 mL) compared to the filtration-based methods (15 to 45 mL) which may be co-concentrated.

### 4.2. Viral Quantification

PEG was not assessed for norovirus GI and GII quantification due to lack of nucleic acid extract volume, as was the case for SARS-CoV-2 WGS and norovirus typing. VS provided the greatest viral concentrations with increases of 83.2-, 19.5-, 13.5-, 7.9- and 7.2-fold for SARS-CoV-2, GI, GII, phi6 and crAssphage compared to AS (Figure 2). SARS-CoV-2 detection rates were high for all methods but lowest for AS at 84.75% (Supplementary Table S1). The increased detection rates for IP over VS for SARS-CoV-2 equates to an additional 6 positive samples out of 236 (2.6%), indicating increased sensitivity for detecting SARS-CoV-2. For GI and GII, 100% detection rates were seen for all methods under investigation. High rates of detection were expected due to those previously observed for norovirus and SARS-CoV-2 in wastewater in England across similar time periods [48,61].

Assessment of the target copies/L and detection rates alone are not sufficient for determining method performance. The former indicates target recovery (without sample spiking) and method accuracy in terms of its closeness to endogenous viral RNA concentrations. The latter indicates sensitivity but should be used in conjunction with RT-qPCR Cq to guide judgements on assay sensitivity irrespective of the starting wastewater volume. For SARS-CoV-2, the copies/L data were mirrored by the Cqs, while for norovirus GI and GII, AS provided the lowest copies/L and IP showed the highest Cqs (Figure 2D,E and Figure 3B,C). This indicates IP may run into sensitivity issues before AS. Further investigation into the method sensitivity and limits of detection, however, should be performed for all target pathogens. In addition, method variability should be further investigated as it has been reported that centrifugal concentrator (CC) methods show increased levels of variability [23,24,32].

Although many previous studies have explored the impact of viral concentration method on quantification of SARS-CoV-2, we will only discuss those that use ≥two methods comparable to those implemented here. The majority used PEG and a CC method with four of seven reporting that PEG outperformed the CC. The first reported PEG (500 mL) recovered 2.83% (OC43) while the CC recovered only 0.8% [19]. Sampling disparity, however, makes comparison difficult with 4 samples for PEG and 22 for CC. Overall, SMF (250 mL) produced the best recovery at 4.6%. The second study reported PEG (30 mL) showed greater recovery (≈9%) and lower Cq (≈23.5) for SARS-CoV-2 compared to the CC at ≈7% and 24.0, respectively [62]. Ultracentrifugation (30 mL), however, performed best with ≈24% recovery and the lowest Cq at ≈22.25. The third study reported better recovery for PEG (50 mL) compared to CC (50 mL) at 0.1% and 0.008%, respectively [32]. An increase in SARS-CoV-2 concentration for PEG at ≈4.9 log10 copies/L compared to ≈4.5 log10 copies/L for CC was also reported. Their direct extraction method (50 mL) provided the highest recovery (2.1%) and concentrations (5.25 log10 copies/L). The final study observed that PEG improved SARS-CoV-2 recoveries (59.4% to 63.7%) compared to CC (33.0% to 42.6%), both processing 40 mL [20].

The remaining three studies reported improved CC performance over PEG. The first found CC outperformed PEG with murine hepatitis virus recovery at 56% compared to 44%, both processing 50 mL [17]. An electronegative membrane-based approach, however, showed the best recovery at 65.7%. The second study reported increased recovery of bovine coronavirus at 0.36% for CC (50 mL) compared to 0.08% PEG (200 mL) [30]. SARS-CoV-2 concentrations were also higher (≈3.75 log10 copies/L) for CC compared to PEG (≈3.25 log10 copies/L). An electronegative membrane-based approach was reported to provide the greatest sensitivity. The third study used two ultrafiltration methods and three precipitation methods to quantify 12 viruses [23]. Independent of the viral target, recovery was greatest for the CC method (12.2%) followed by PEG-beef gelatine (10.9%), IP (5.1%), AS (5.0%) and PEG (2.3%). This supports the overall trends for both SARS-CoV-2 and norovirus GI and GII in this study. When considering the targets independently, the PEG-beef gelatine method performed best for SARS-CoV-2 and GII.

### 4.3. Norovirus Dual-Typing Using Long Amplicon Sequencing

VS performed best for GI and IP for GII (Figure 4). Read quality was not significantly impacted by viral concentration method, although IP and VS outperformed AS (Figure 4A). For GI, there was a large increase in aligned reads with VS producing 1.3- and 2.6-fold higher read counts than IP and AS (Figure 4E). Although IP showed an increase in aligned reads for GII, the performance gains were small at 13.5% and 16.0% from VS and AS when compared to the gains in performance for GI for VS at 30.0% and 62.5% increases from IP and AS. Similar observations were made for taxa richness where the performance gains for GI VS were greater (30.2% and 159.2% higher compared to IP and AS) than those for GII IP (16.6% and 20.7% compared to VS and AS) (Figure 4G,H).

The improved performance of IP over VS for GII was not seen in the RT-qPCR data (Figure 2 and Figure 3). This could be due to an increased ability of VS to capture free or fragmented nucleic acids <1 kb which were quantified during RT-qPCR. The increased impact of concentration method for GI may be due to its lower titres in wastewater averaging 3.8-fold lower than GII (Figure 2A,B). This may indicate VS has an increased recovery efficacy, which is supported by the quantification of GI nucleic acids 1.7- and 2.3-fold greater than AS and IP (Figure 3B). These observations highlight the importance of re-assessing WBS’ alignment with clinical data when a change in viral concentration methodology has occurred, as an increased capture of free or fragmented nucleic acids could lead to data inaccuracies.

### 4.4. SARS-CoV-2 Long Amplicon Whole-Genome Sequencing

Coverage and read depth were low, with only VS achieving coverage over 80% for two samples (Figure 5). Low median read qualities (12.8 to 13.7), high levels of non-specific amplification (Supplementary Figure S2) and potentially fragmented nucleic acids are likely to have led to data loss and impact assay sensitivity. An increase in coverage (R = −0.52, *p* = 0.003) and aligned reads (R = −0.40, *p* = 0.027) was seen as Cq reduced (Supplementary Figures S3 and S4), indicating improved recovery of nucleic acids will increase aligned reads and genome coverage as expected.

Overall, VS showed higher median read qualities and increases in aligned reads (4.2- and 2.2-fold), average read depth (13.2- and 12.0-fold) and coverage (2.2- and 6.4-fold) when compared to AS and IP (Figure 6). VS was, therefore, the best performing method for long amplicon SARS-CoV-2 WGS. The improved performance may indicate increased recovery of SARS-CoV-2 particles and RNA ≥ 1200 bp in length. The influence of PCR inhibitors, however, should not be ignored, as the higher levels identified for AS during one-step RT-qPCR are likely to be indicative of those on impacting reverse transcription and PCR efficiency during library preparation.

No previous research has studied the impact of viral concentration technique on long amplicon sequencing of SARS-CoV-2, but two studies have used short amplicons. The first compared two PEG methods with varying starting volumes to a Nanotrap method [28]. WGS using NimaGen EasySeq RC-PCR paired with Illumina 2 × 150 bp reads showed an increased number of variants, genome depth (≈1.7-fold) and aligned bases (≈2.3-fold) along with an improved QC pass rate (>≈3.6-fold) for PEG 150 mL compared to PEG 37.5 mL and Nanotrap. The second study investigated the impact of aluminium-based adsorption and direct capture on sequencing using the ARTIC panel V3 and V4 primers with Illumina 2 × 200 bp reads [33]. For the V4 primers, direct capture increased the mean number of SARS-CoV-2 reads (20.5% to 55.1%), genome coverage (83.7% to ≈55.0%) and genome depth (727× to 400×) when compared to the adsorption-based method.

The lack of methodological overlap with previous studies impedes the ability to build a broader picture of the impact of viral concentration methods on sequencing. It does, however, highlight the importance of assessing viral concentration methods’ impact on sequencing. This appears to be especially important for PCR-based sequencing, as it has been shown here and in previous publications that viral concentration method can have a large impact on sequencing data quality and quantity [28,33].

### 4.5. Overall Method Performance

Overall, VS performed best for quantification and sequencing with a score of 67 out of a maximum of 78 compared to IP and AS at 53 and 42 when applying a rank-based scoring system (Supplementary Table S5). The small performance gains observed for IP over VS for norovirus GII sequencing are outweighed by the gains for VS across RT-qPCR inhibition, viral quantification and sequencing of GI and SARS-CoV-2. Implementing the VS method would likely improve analytical sensitivity evidenced through reductions in Cq representing increased levels of purified viral RNA. VS may also provide increased accuracy of viral quantification and show better performance for low-titre viruses, as discussed for norovirus GI.

The improved performance of VS is likely due to improved efficacy of ultrafiltration for concentrating viral particles and nucleic acids when compared to precipitation-based techniques. Furthermore, the total surface area that viral particles or free nucleic acids are concentrated onto may also influence recovery. VS sample concentration occurs in a self-contained area with a surface area of ≈6.8 cm^2^ while AS and PEG have a total container surface areas of ≈308 cm^2^, potentially reducing the probability of recovery following precipitation. Further investigation into the surface area covered by precipitants, however, should be explored.

The container surface area/wastewater volume ratios and the materials used to manufacture containers may also influence viral particle and nucleic acid adsorption to plasticware. These ratios are greater for VS (3.3 cm^2^/mL) than for AS or PEG (1.8 cm^2^/mL) and may contribute towards the reported higher levels of variability for CC methods. This may be resolved though the effective blocking of plasticware with pre-treatments or by spiking samples with bovine serum albumin or gelatine.

Although this study has helped build the breadth and depth of information available on viral concentration techniques employed in WBS, the performance of the different methods across studies has shown to be variable, as previously discussed. This is likely down to differences in specific protocols, sample volumes being processed, the materials used in the manufacture of consumables, the wastewater samples undergoing investigation, experimental design and the areas of technical expertise in the labs performing the analysis. As wastewater surveillance systems continue to be built and adapted to different pathogens, continued method optimisation should be performed to ensure the quality of data being produced and used for WBS.

## Figures and Tables

**Table 1:** The bioinformatic packages and parameters used for processing the SARS-CoV-2 long amplicon whole-genome sequencing data.

Procedure	Package	Parameter
Split reads [50]	duplex_tools version 0.2.14	duplex_tools split_on_adapter --threads 12 --allow_multiple_splits Native
Trim primers [51]	cutadapt version 3.4	cutadapt -j12 --action=trim -n 1 -e 0.30 -O 12 –revcomp -g file:primer_sequences.fasta --discard-untrimmed
Filter by quality [52]	Seqkit version 2.3.0	seqkit seq -Q 20
Filter by length [52]	Seqkit version 2.3.0	seqkit seq -m900 -M 1500
Map reads [53]	Minimap version 2.24	minimap2 -ax lr:hq
Sort and convert SAM files to BAM [54]	Samtools version 1.13	samtools sort -o .bam
Index BAM files [54]	Samtools version1.13	samtools index
Calculate read depth [55]	IGV version 2.17.2	count -w 100

## Data Availability

The processed SARS-CoV-2, norovirus, phi6 and crAssphage qPCR and RT-qPCR inhibition data are available in Supplementary Data S1 and S2. Norovirus and SARS-CoV-2 sequencing data is available at BioProject PRJNA1177646.

## Supplementary Materials

The following supporting information can be downloaded at: https://www.mdpi.com/article/10.3390/microorganisms13020229/s1. Supplementary Methods S1: The crAssphage qPCR methods adapted from Stachler, E.; Bibby, K. Metagenomic evaluation of the highly abundant human gut bacteriophage crAssphage for source tracking of human fecal pollution. Environ. Sci. Technol. Lett. 2014, 1 (10), 405-409. Supplementary Data S1: qPCR and RT-qPCR inhibition data from wastewater nucleic acid extracts analysed at Cefas, Weymouth where SARS-CoV-2 RT-qPCR inhibition was measured and norovorius GI and GII concentrations were calculated. Samples were processed with different viral concentration methods ammonium sulphate (AS), concentrating pipette (IP), polyethylene glycol (PEG) and Vivaspin (VS). N = 20. Supplementary Data S2: qPCR data from wastewater samples analysed by the Environment Agency including SARS-CoV-2, crAssphage and phi6 process control. Samples were processed with different viral concentration methods ammonium sulphate (AS), concentrating pipette (IP), polyethylene glycol (PEG) and Vivaspin (VS). N = 240. Supplementary Table S1: SARS-CoV-2 and norovirus GI and GII RT-qPCR detection rates for samples processed using the ammonium sulphate (AS), concentrating pipette (IP) and Vivaspin (VS) viral concentration methods. Supplementary Table S2: GI taxa detected for samples processed using ammonium sulphate (AS), InnovaPrep (IP) and Vivaspin (VS) viral concentration techniques. N = 10. Supplementary Table S3: GII taxa detected for samples processed using ammonium sulphate (AS), InnovaPrep (IP) and Vivaspin (VS) viral concentration techniques. N = 10. Supplementary Table S4: SARS-CoV-2 PCR tiling-based WGS amplicon depth following negative control background adjustments for samples processed using the ammonium sulphate (AS), concentrating pipette (IP) and Vivaspin (VS) viral concentration methods. Supplementary Table S5: Method performance assessment table for samples processed using the ammonium sulphate (AS), concentrating pipette (IP) and Vivaspin (VS) viral concentration methods. Supplementary Figure S1: SARS-CoV-2 concentrations from a sub-selection of samples before (A) and after (B) RT-qPCR inhibition correction. Samples were processed with different viral concentration methods ammonium sulphate (AS), concentrating pipette (IP), polyethylene glycol (PEG) and Vivaspin (VS). P-values reported are from the initial hypothesis testing. Groups within a subfigure sharing the same lettering are not significantly different from each other following pairwise comparison (*p* > 0.05). N = 17. Supplementary Figure S2: TapeStation results for SARS-CoV-2 WGS showing the negative controls from PCR pool 1 and 2 in lanes B1 and C1. The remaining lanes are; lanes D1, E1 and F1 are from sample 1, G1, H1 and A2 from sample 2, B2, C2 and D2 from sample 3, E2, F2 and G2 from sample 3; all of which are pool 1 PCR products from the ammonium sulphate, concentrating pipette and Vivaspin methods, respectively. Supplementary Figure S3: Correlation between SARS-CoV-2 RT-qPCR Cq and WGS genome coverage for samples processed using the ammonium sulphate (AS), concentrating pipette (IP) and Vivaspin (VS) viral concentration methods. Supplementary Figure S4: Correlation between SARS-CoV-2 RT-qPCR Cq and the number of aligned reads for samples processed using the ammonium sulphate (AS), concentrating pipette (IP) and Vivaspin (VS) viral concentration methods.
